# Performance of a quality assurance program for assessing dental health in methamphetamine users

**DOI:** 10.1186/s12903-015-0057-z

**Published:** 2015-07-05

**Authors:** Bruce A. Dye, Lauren Harrell, Debra A. Murphy, Thomas Belin, Vivek Shetty

**Affiliations:** National Institute of Dental and Craniofacial Research, National Institute of Health, Bethesda, MD USA; Integrated Substance Abuse Programs, University of California Los Angeles, Los Angeles, CA USA; Department of Biostatistics, Fielding School of Public Health, University of California Los Angeles, Los Angeles, CA USA; Oral & Maxillofacial Surgery, School of Dentistry, University of California Los Angeles, Los Angeles, CA USA

**Keywords:** Dental caries, Methamphetamine use, Reliability, Quality assurance, NHANES

## Abstract

**Background:**

Systematic characterization of the dental consequences of methamphetamine (MA) abuse presupposes a rigorous quality assurance (QA) program to ensure the credibility of the data collected and the scientific integrity and validity of the clinical study. In this report we describe and evaluate the performance of a quality assurance program implemented in a large cross-sectional study of the dental consequences of MA use.

**Methods:**

A large community sample of MA users was recruited over a 30 month period during 2011–13 and received comprehensive oral examinations and psychosocial assessments by site examiners based at two large community health centers in Los Angeles. National Health and Nutrition Examination Survey (NHANES) protocols for oral health assessments were utilized to characterize dental disease. Using NHANES oral health quality assurance guidelines, examiner reliability statistics such as Cohen’s Kappa coefficients and inter-class correlation coefficients were calculated to assess the magnitude of agreement between the site examiners and a reference examiner to ensure conformance and comparability with NHANES practices.

**Results:**

Approximately 9 % (*n* = 49) of the enrolled 574 MA users received a repeat dental caries and periodontal examination conducted by the reference examiner. There was high concordance between the reference examiner and the site examiners for identification of untreated dental disease (Kappa statistic values: 0.57–0.75, percent agreement 83–88 %). For identification of untreated caries on at least 5 surfaces of anterior teeth, the Kappas ranged from 0.77 to 0.87, and percent agreement from 94 to 97 %. The intra-class coefficients (ICCs) ranged from 0.87 to 89 for attachment loss across all periodontal sites assessed and the ICCs ranged from 0.79 to 0.81 for pocket depth. For overall gingival recession, the ICCs ranged from 0.88 to 0.91. When Kappa was calculated based on the CDC/AAP case definitions for severe periodontitis, inter-examiner reliability for site examiners was low (Kappa 0.27–0.67).

**Conclusion:**

Overall, the quality assurance program confirmed the procedural adherence of the quality of the data collected on the distribution of dental caries and periodontal disease in MA-users. Examiner concordance was higher for dental caries but lower for specific periodontal assessments.

## Background

The initial reported association of methamphetamine (MA) abuse with advanced dental disease [1] engendered a slew of case reports describing the severe dental consequences encountered in MA users [2–8]. Popularly known by the moniker “meth mouth,” the examples of extreme dental decay and tooth loss were disseminated widely by the news media without being linked to rigorous epidemiological studies that validated the reports. As a first step towards scientifically verifying the accumulating anecdotal evidence, we had previously utilized the infrastructure of a large multisite clinical study (Methamphetamine Treatment Project or MTP) to systematically examine the health consequences of chronic MA use in a representative sample of users [9]. Comprehensive medical and brief dental assessments carried out by participating physician examiners were used to define the nature and rates of medical and dental disease in a subset of 301 MA users. The dental findings were compared to the dental status of a sociodemographically similar group of non-MA using participants enrolled in the National Health and Nutrition Examination Survey (NHANES III). One of our main findings was that overt dental disease was a key distinguishing comorbidity in chronic MA users [9]. The MA users from the MTP cohort had significantly higher rates of missing teeth and dental disease than demographically comparable controls and reported long-term unmet oral health needs.

Building on our precursory findings, we conducted a follow-up, cross-sectional study comprising a new cohort of MA users from the community and involving detailed dental examinations by trained dentists following protocols aimed at achieving well-calibrated assessments. Primary goals were to characterize the occurrence and nature of dental caries and periodontal disease in individuals with a range of MA-use behaviors and to investigate whether the specificity of the caries patterns could be used to distinguish MA users from non-users. Because the oral health assessments involved different raters at separate collection sites, ensuring the comparability and uniformity of the data being collected was a critical prerequisite. Take for example the consequences of inter-rater variability on inferences based on periodontal assessments. Variations in pocket depths tend to be relatively small (measured in millimeters) and as such, even slightly imprecise assessments resulting from procedural errors by the dental examiners could generate substantial bias and have an adverse impact on the validity of corresponding inferences. Thus, an effective and robust Quality Assurance (QA) program is essential for standardized data collection across sites and for monitoring and ensuring the precision and accuracy of the data collected. This paper provides an overview of the QA program used for our cross-sectional study of the dental consequences of MA use, with particular attention to quality-control procedures. A main objective of the paper is to assess the performance of the QA program by examining the inter-rater reliability or agreement between the field dental examiners and the reference examiners for individual data elements.

## Methods

### Study setting

The study was conducted in Los Angeles County, one of the largest and most populous urban areas in the USA and beset with high rates of MA use [10, 11]. Between February 9, 2011 and August 26, 2013, 574 MA users recruited from local communities underwent comprehensive oral examinations and psychosocial assessments at dental clinics associated with two large community health centers: a) the AIDS Project, Los Angeles (APLA) center that primarily serves a sociodemographically diverse group of individuals with HIV/AIDS, and b) the Mission Community Hospital (Mission) in the San Fernando Valley that caters to a large, underserved migrant population. The study sites were chosen to provide access to a diverse cohort of afflicted Angelenos with a broad range of MA-use behaviors. For the main study, we used a case-control study design that compared the MA users to a control group of propensity-score matched samples from the National Health and Nutrition Examination Survey (NHANES) [12, 13], where propensity scores were used to identify a demographically comparable subset of individuals from the population-based NHANES. Study findings presented in this paper are based on a selected sub-sample of participants who underwent replicate examinations for data quality assurance purposes. The study design and data collection protocol was reviewed and approved by the UCLA Institutional Review Board. Trained dental examiners conducted the dental examinations, with the data recorded by dental assistants. In conjunction with the dental exam, an experienced bilingual interviewer conducted comprehensive assessments of psychosocial, behavioral, and substance-use characteristics of study participants.

### Training and quality assurance

The study was launched with a 2-day project orientation for all persons involved in the study, including the Principal Investigator, co-investigators, project manager, reference examiners, dental examiners, dental assistants, research assistant, and the statistical and data management team. At this initial meeting, the lead investigators reviewed the study’s scientific background, objectives, inclusion and exclusion criteria, evaluation criteria, key outcome variables and study enrollment targets. The practical aspects of the study were discussed including recruitment and screening strategies, management of any adverse events, technical considerations relating to data collection and recording, as well as responsibilities with regard to protocol adherence, quality assurance and logistical support.

Training and Quality Assurance (QA) were conducted under the leadership of BD (lead reference examiner), who is the national trainer and reference examiner for NHANES. He was assisted by a locally-based dental epidemiologist (local reference examiner) who provided ongoing monitoring of the dental examiners, evaluating their assessments and providing remediation when necessary. A dentist from each participating health center was selected and trained to conduct a standardized dental examination. Both dentists provided direct patient care at the participatory sites but had little prior experience in clinical research. On the 1st day of the orientation meeting, the lead reference examiner used lecture and slide presentations to familiarize the study dental examiners with customary NHANES study protocols and assessment criteria followed by explanation of the examination technique and equipment use. To initiate the standardization process, demonstration examinations, conducted by the lead reference examiner, was followed by the trainee dental examiners conducting practice examinations in volunteer subjects. During the standardization process, the trainee examiners were encouraged to ask questions regarding assessment criteria while conducting the study protocols. In an effort to minimize differences in examination findings, data from each standardization round were reviewed for inconsistencies and findings were discussed with the trainee examiners led by the reference examiner. In a subsequent calibration phase, the reference examiner and dental examiners repeated dental and periodontal assessments in the same set of 12 volunteers.

Once the field study was initiated, the local reference examiner visited each site monthly to observe data collection and to randomly replicate the dental examination in 2–3 subjects. Data from these replicate exams were used to produce inter-rater reliability statistics to evaluate examiner performance and to provide feedback. If an examiner’s performance fell below an acceptable level, retraining was conducted on site. About 1 year after study launch, the lead reference examiner returned to observe field operations and, along with the local reference examiner, conducted another round of calibration exercises with the dental examiners. The calibration visits took place at each clinical site during normal field operations and involved 12 subjects enrolled in the study. The monitoring visits helped determine whether the dental examiners were conducting the oral health examinations within the parameters of the NHANES study protocols and if the standards for examination between the dental examiners and reference examiner had been maintained.

The project manager oversaw the data collection activities at each field site, providing ongoing logistical support and supervision and ensuring that the each site followed all appropriate procedures for subject recruitment and consenting. Dental examiners were clinic-based for this study. At the APLA site, where the bulk of the assessments took place, the study examiner remained for the duration of the study. The initial examiner at Mission site departed towards the midpoint of the data collection phase, and a replacement examiner was trained and calibrated to collect data. Overall, 51 enrolled subjects, representing 9 % of the study sample, received replicate exams.

### Variables and their measurement

The main oral-health outcome variables were the rates and patterns of dental caries and the periodontal disease status of the subjects. Assessments for dental caries and periodontal status adhered to NHANES examination protocols, which have been described in greater detail elsewhere [14–16]. In brief, NHANES diagnostic criteria were used to assess for dental caries at the surface-level and presence required manifest cavitation. Examinations were conducted under artificial light with the study participant in the supine position using a standard explorer and dental mirror without additional magnification. Radiographs were not taken. A Hu-Friedy periodontal probe color-coded and graduated at 2–4–6–8–10–12 mm was used to assess periodontal status. Dental caries experience was calculated as the number of diseased (D), missing (M), and filled (F) teeth (T) and the number of untreated dental caries was calculated as the number of diseased surfaces (DS). Periodontal disease status was assessed using the case definitions recommended for periodontitis surveillance by the CDC Periodontitis Workgroup (CDC/AAP) ([17]. The CDC/AAP case definitions require information from two interproximal sites (DF, MF, ML, and/or DL) and are not dependent upon the presence of an adjacent tooth. Gingival recession and pocket depth measures were made at four sites per tooth (the disto-facial (D), mid-facial (B), mesio-facial (M), and the disto-lingual (DL) sites). An algorithm calculated loss of attachment from the information on gingival recession and pocket depth. All four quadrants were examined and 3rd molars were excluded.

### Data collection and management

To ensure standardization and quality assurance in data collection and processing, all dental and psychosocial data were captured directly on a laptop computer using a web-based data-management system developed and maintained by the UCLA-Semel Institute Statistics Core (SIStat). Data collected through the user-friendly graphical interface on the laptop was encrypted and transmitted to be stored centrally in a secure server with firewall protection. Built-in logic and data-range checks allowed data verification to prevent invalid data. Automated reports and dashboards allowed the investigators and project manager to monitor the quality of the data collected at each clinical site by generating a variety of summary reports on data completeness and questionable values. The real-time input verification facilitated the timely identification and resolution of any problems in data collection and processing.

### Statistical methods

SAS software (Version 9.3; SAS Institute Inc., Cary, NC, USA) was used for statistical analysis and data handling. Demographic information was tabulated for the full sample (*n* = 574) as well as within each of the replicate samples at APLA (*n* = 33) and Mission (*n* = 18). Participants who indicated that they had used methamphetamine for less than 10 of the last 30 days at the time of screening were classified as being “light” methamphetamine users, while the study participants who used MA for more than 10 of the last 30 days were classified as “moderate +” users. Education level was broken into three categories: less than high school, high school completion, and more than high school. High school completion was indicated by high school graduation or by obtaining a GED.

The reliability analysis was conducted using a set of both continuously-scaled and dichotomous outcome measurements. Continuously-scaled measurements from the caries examination included DMFT (the total number of decayed, missing, or filled teeth in a participant’s mouth), DFT (number of decayed or filled teeth), DS (number of decayed surfaces), and tooth retention (number of teeth present in the mouth). Continuously-scaled outcomes from the periodontal examination included mean gum recession, pocket depth, and calculated attachment loss per participant. Average attachment loss and pocket depth were additionally stratified by the four periodontal sites (mid-facial, mesial-facial, disto-facial, disto-lingual), recognizing that different site types could have differing variability. Means and standard errors were produced for each rater on these outcomes, as well as the bias, defined as the difference between the examination rater measurement and the reference measurement. Intra-class correlation (ICC) coefficients were estimated as the primary reliability statistic for the continuously-scaled measurements. The intra-class correlation was defined as the ratio of variance between subjects to the total variance between and within subjects. ICCs closer to 100 % indicate greater inter-rater reliability.

Discrete outcomes from the caries examination contained the caries experience (defined above), having at least one surface of untreated decay, having at least one restoration or surface restoration, tooth retention (all teeth present), and having at least five anterior surfaces with untreated decay. From the periodontal examination, the dichotomous outcomes included having at least one site with attachment loss greater than or equal to a given threshold (taken to be either 3, 4, or 6 mm); having at least one site with pocket depth greater than or equal to a given threshold (either 4, 5, or 7 mm); and indicators of periodontitis, moderate periodontitis, and severe periodontitis. Percent agreement between the examination and reference raters was computed as one metric of reliability. Cohen’s kappa statistic and a corresponding asymptotic standard error (SE) were also produced. Examiner strength of agreement using the Kappa coefficient was not evaluated by hypothesis (statistical) testing and instead was evaluated by applying commonly used guidelines [18].

## Results

Table 1 shows the number of persons examined in the main study and those receiving a repeat examination by the reference examiner by select characteristics. Study participants were generally older, male, Hispanic or non-Hispanic black, had lower educational attainment, were smokers, or were moderate/heavy methamphetamine users. Overall, the distribution of demographic and behavioral characteristics for individuals participating in the replicate exams was similar to that observed in the main study.

**Table 1:** Sociodemographic characteristics of methamphetamine users

	Sample size		
	Number (%)	Site A replicates	Site B replicates
Age			
<30 years	48 (8.3 %)	3 (9.1 %)	2 (11.1 %)
≥30 years	529 (91.7 %)	30 (90.9 %)	16 (88.9 %)
Sex			
Male	465 (80.6 %)	22 (66.7 %)	16 (88.9 %)
Female	112 (19.4 %)	11 (33.3 %)	2 (11.1 %)
Race/ethnicity			
Hispanic	179 (31.0 %)	8 (24.2 %)	2 (11.1 %)
Non-Hispanic Black	244 (42.3 %)	19 (57.6 %)	9 (50.0 %)
Non-Hispanic White	111 (19.2 %)	6 (18.2 %)	6 (33.3 %)
Education			
Less than HS	173 (30.0 %)	11 (33.3 %)	3 (16.7 %)
HS	201 (34.8 %)	10 (30.3 %)	8 (44.4 %)
More than HS	203 (35.2 %)	12 (36.4 %)	7 (38.9 %)
Smoking History			
Current smoker	397 (68.9 %)	24 (72.7 %)	17 (94.4 %)
Former smoker	55 (9.6 %)	3 (9.1 %)	0 (0 %)
Never smoked	124 (21.5 %)	6 (18.2 %)	1 (5.6 %)
Methamphetamine Use			
Light	256 (44.4 %)	12 (36.4 %)	7 (38.9 %)
Moderate/ Heavy	321 (55.6 %)	21 (63.6 %)	11 (61.1 %)
Total	574	33	18

The inter-rater reliability statistics by clinic examination site (A & B) for categorical evaluations of dental caries and periodontal status are shown in Table 2. The Kappa statistics for untreated dental caries ranged from 0.57 to 0.75, with percent agreement ranging from 83 to 88 %. For identifying untreated caries on at least 5 surfaces of anterior teeth, Kappa scores were 0.77 and 0.87, and percent agreement was 94 and 97 %. Examiners at both clinical sites performed equally when identifying caries experience (Kappa 1.00 and percent agreement 100 %). Kappa scores for various thresholds of pocket depth ranged from 0.20 to 0.77 and for attachment loss ranged from 0.29 to 0.52 (among Kappa scores that could be computed, as Kappa statistics could not be estimated when the probability of classification into a given category for one or both raters is 0 or 100 %). There were three instances where the attachment loss thresholds were identified in 100 % of the subjects by one or both raters, and thus the Kappa statistics could not be computed. The percent agreements for these three instances ranged between 72.2 and 100 %. When Kappa was calculated based on the CDC/AAP case definitions for moderate and severe periodontitis, inter-examiner reliability was higher at site B compared to site A. For severe periodontitis, the Kappa was 0.27 for site A, whereas at site B the reliability statistic was calculated at 0.67.

**Table 2:** Inter-rater reliability statistics for selected dental health conditions

Site	Assessment	*n*	Ref case %	Examiner case %	Percent agreement	Kappa	-Kappa)
	Dentition/Dental caries						
A	Tooth Retention (has all 28 teeth excluding 3rd molars)	33	9.1 %	9.1 %	100 %	1.00	0
B		18	22.2 %	22.2 %	100 %	1.00	0
A	Has Untreated Caries (DT ≥ 1)	33	57.6 %	57.6 %	87.9 %	0.75	0.12
B		18	83.3 %	83.3 %	83.3 %	0.57	0.20
A	Has Restorations (FT ≥ 1)	33	84.9 %	84.9 %	100 %	1.00	0
B		18	83.3 %	83.3 %	100 %	1.00	0
A	Caries experience (DMFT >0)	33	93.9 %	93.9 %	100 %	1.00	0
B		18	100 %	100 %	100 %	NA^a^	NA^a^
A	At least 5 surfaces of anterior decay (DS ≥ 5)	33	12.1 %	15.2 %	97.0 %	0.87	0.13
B		18	11.1 %	16.7 %	94.4 %	0.77	0.22
A	Has root caries	33	39.4 %	48.5 %	90.9 %	0.82	0.10
B		18	33.3 %	27.8 %	83.3 %	0.61	0.20
	Periodontal Status						
A	At least one site with AL ≥3	31	96.8 %	100 %	96.7 %	NA^a^	NA^a^
B		18	100 %	100 %	100 %	NA^a^	NA^a^
A	At least one site with AL ≥4	31	87.1 %	90.3 %	90.3 %	0.52	0.24
B		18	72.2 %	100 %	72.2 %	NA^a^	NA^a^
A	At least one site with AL ≥6	31	51.6 %	51.6 %	64.4 %	0.29	0.17
B		18	72.2 %	61.1 %	77.9 %	0.51	0.21
A	At least one site with PD ≥4	31	90.3 %	87.1 %	83.9 %	0.20	0.24
B		18	88.9 %	77.8 %	88.9 %	0.61	0.24
A	At least one site with PD ≥5	31	48.4 %	45.2 %	77.4 %	0.55	0.15
B		18	61.1 %	61.1 %	88.9 %	0.77	0.16
A	At least one site with PD ≥7	31	12.9 %	3.2 %	90.3 %	0.37	0.27
B		18	11.1 %	11.1 %	88.9 %	0.44	0.33
A	At least one site with bleeding	31	64.5 %	83.9 %	61.3 %	0.04	0.16
B		14	92.9 %	92.9 %	100 %	1.00	0
A	Total Periodontitis	31	93.6 %	90.3 %	90.3 %	0.35	0.29
B		18	100 %	83.3 %	83.3 %	NA^a^	NA^a^
A	Severe Periodontitis	31	29.0 %	35.5 %	67.7 %	0.27	0.18
B		18	55.6 %	50 %	83.3 %	0.67	0.23
A	Moderate Periodontitis	31	61.3 %	51.6 %	58.2 %	0.15	0.17
B		18	44.4 %	28.8 %	72.2 %	0.42	0.21

*n* number of replicate exams, *SE* asymptotic standard error of Kappa, *DT* decayed teeth, *FT* restored/filled teeth, *DMFT* decayed, missing, filled teeth, *DS* decayed tooth surfaces, *AL* attachment loss, *PD* pocket depth, Periodontitis defined using CDC/AAP case definitions

NA^a^ -Kappa statistics could not be computed when any frequencies in a 2 × 2 table of raters and outcomes are zero. When the percent of subjects rated with an outcome was 100 %, Kappa statistics were not obtained

Table 3 shows the mean values and intra-class correlation coefficients when continuous measures of periodontal status and dental caries were used. For overall attachment loss (AL) and pocket depth (PD) across all four periodontal sites, the intra-class coefficients ranged from 0.87–0.89 and 0.79–0.81 respectively. For measures of overall gingival recession (CJ mean), the ICCs ranged from 0.88 to 0.91. For attachment loss, ICC for facial (B) measures ranged from 0.54 to 0.82 and for the disto-lingual (DL) measures from 0.64 to 0.87. For pocket depth, the ICC values were lower than for attachment loss at the B site. The ICCs for interproximal facial and disto-lingual pocket depth measures ranged from 0.43 to 0.60 and 0.75 to 0.85 respectively. When looking at caries experience and tooth retention, the dental examiners at both sites A and B demonstrated nearly ideal correlation with ICCs ranging from 0.96 to 0.99.

**Table 3:** Dental examiner inter-class correlation coefficients (ICCs) for selected periodontal measures

Site	Assessment	*n*	Ref Mean (SE)	Examiner Mean (SE)	Bias (SE)	ICC
	Dentition / Dental Caries					
A	Mean DMFT	33	13.06 (1.28)	13.27 (1.28)	0.21 (0.17)	0.99
B		18	13.00 (1.82)	13.33 (1.86)	0.33 (0.23)	0.99
A	Mean DFT	33	8.33 (0.99)	8.55 (0.97)	0.21 (0.17)	0.98
B		18	7.61 (1.20)	7.50 (1.11)	−0.11 (0.29)	0.97
A	Mean DS	33	9.18 (3.12)	8.89 (3.11)	−0.30 (0.27)	0.99
B		18	9.00 (3.94)	7.55 (3.10)	−1.44 (1.00)	0.96
A	Mean number of teeth present	33	23.27 (1.14)	23.27 (1.14)	0.00 (0.04)	0.99
B		18	22.28 (1.62)	22.06 (1.61)	−0.22 (0.13)	0.99
	Periodontal Status					
A	Mean AL	31	2.39 (0.21)	2.51 (0.20)	0.12 (0.09)	0.89
B		18	2.92 (0.22)	2.80 (0.21)	−0.12 (0.11)	0.87
A	Mean PD	31	2.50 (0.08)	2.50 (0.09)	0.00 (0.29)	0.81
B		18	2.65 (0.12)	2.47 (0.14)	−0.18 (0.08)	0.79
A	Mean Recession	31	0.11 (0.18)	−0.02 (0.15)	−0.13 (0.08)	0.88
B		18	−0.27 (0.19)	−0.34 (0.20)	−0.06 (0.09)	0.91
A	Mean AL-B sites	31	2.37 (0.20)	2.57 (0.20)	0.20 (0.11)	0.82
B		18	2.48 (0.15)	2.07 (0.15)	−0.41 (0.12)	0.54
A	Mean AL-M sites	31	2.19 (0.22)	2.10 (0.21)	−0.08 (0.74)	0.81
B		18	2.93 (0.24)	2.97 (0.21)	0.04 (0.14)	0.83
A	Mean AL-D sites	31	2.42 (0.22)	2.58 (0.22)	0.16 (0.10)	0.88
B		18	2.97 (0.29)	3.08 (0.27)	0.11 (0.12)	0.91
A	Mean AL-DL sites	31	2.58 (0.24)	2.81 (0.20)	0.23 (0.11)	0.87
B		18	3.30 (0.32)	3.12 (0.27)	−0.17 (0.26)	0.64
A	Mean PD-B sites	31	1.83 (0.07)	1.96 (0.08)	0.13 (0.06)	0.60
B		18	1.86 (0.09)	1.54 (0.09)	−0.32 (0.07)	0.43
A	Mean PD-M sites	31	2.73 (0.10)	2.73 (0.11)	0.003 (0.06)	0.81
B		18	2.85 (0.15)	2.84 (0.13)	−0.01 (0.09)	0.81
A	Mean PD-D sites	31	2.80 (0.09)	2.77 (0.10)	−0.03 (0.07)	0.74
B		18	2.96 (0.16)	2.79 (0.17)	−0.17 (0.08)	0.84
A	Mean PD-DL sites	31	2.63 (0.11)	2.52 (0.10)	−0.11 (0.05)	0.85
B		18	2.91 (0.17)	2.71 (0.20)	−0.20 (0.13)	0.75

*n* number of replicate exams, *ICC* intra-class correlation coeficient, *DMFT* decayed, missing, filled teeth, *DFT* decayed and filled teeth, *DS* decayed tooth surfaces, *AL* attachment loss, *PD* pocket depth, *B* buccal/facial site, *M* mesio-facial site, *D* disto-facial site, *DL* disto-lingual site

## Discussion

Overall, quality assurance findings from this study investigating the distribution of dental caries and periodontal disease in MA-using individuals indicate that substantial agreement existed with the reference examiner and the site examiners for dental caries. However, examiner concordance was lower for specific periodontal assessments. In this study, we focus on two key measures of examiner reliability: intra-class correlation coefficients (ICCs) for continuously-scaled data and Kappa statistics for categorical data. Kappa statistics incorporate a correction for the agreement that would be expected by chance alone. Consequently, their values are lower compared to percent agreement calculations. Even though a number of different standards can be used to ascertain the strength of the agreement between examiners, we have relied on a widely used guideline proposed by Landis and Koch for interpreting kappa scores [18]. In a summary, a kappa statistic ≤ 0 is reflective of having “poor agreement”, > 0 but ≤ 0.20 is “slight agreement”, 0.21–0.40 is “fair agreement”, 0.41–0.60 is “moderate agreement”, 0.61–0.80 is “substantial agreement”, and >0.80 is “almost perfect agreement”.

In this study, examiner percent agreement for selected dentition and dental caries assessments was 83 % or higher and Kappa statistics were 0.77 and higher, indicating substantial to almost perfect agreement between the site examiners with the reference examiner. Nonetheless, agreement among examiners for untreated caries at examination center “Site B” was moderate (0.57). Examiner reliability performance for most of the periodontal assessments ranged from fair to substantial agreement. Overall, examiners from Site A were more in concordance with the reference examiner when assessing dental caries and examiners from Site B were more in concordance with the reference examiner when assessing periodontal disease. Prevalence and bias can influence the calculation of the Kappa coefficient. The greater variance in case prevalence across the sites, the more likely the magnitude of the Kappa statistic can be affected. In our study, participates at Site A were more likely to have more advanced periodontal disease and participants at Site B were more unlikely to have untreated dental caries.

Another set of reliability statistics we have presented in this report are ICCs. Although it has been suggested that a threshold of 0.75 or greater would represent excellent reliability [19], examiner bias (the mean difference between reference examiner and survey examiners) is also an important consideration. Using the conventional measures for caries experience and tooth retention, the examiner reliability for our study should be considered excellent. For examiners at both examination sites, the ICC statistics for DMFT, DFT, DS, and the number of retained teeth were ≥ 0.96. For the periodontal data, the mean attachment loss ICC statistic, as measured for all teeth and eligible sites, was 0.87 and 0.89 for each examiner, indicating excellent overall reliability for AL. When evaluating the ICCs calculated for pocket depth, the range was 0.79 to 0.81. Overall recession measure reliability data also was excellent ranging from 0.88 to 0.91. When evaluating the individual sites, examiner reliability was lower for examiners at Site B compared to Site A at the buccal sites for attachment loss (0.54 vs 0.82) and pocket depth (0.43 vs. 0.60).

It is unclear why examiner reliability was lower for the mid-facial sites given that interproximal sites can be more challenging to accurately measure. It may be that examiners slightly altered the placement of the probe to allow it to slip into a posterior furcation at an angle greater than required, which distorted the actual pocket depth measure. Interestingly, examiner reliability findings from NHANES 2009–10 have indicated a similar issue, with greater variability in examiner reliability at some mid-tooth sites [16]. The current study used the Hu Friedy PCP-12 periodontal probe, which is the same probe used on the current NHANES. The periodontal probes are marked in 2 mm increments, and examiners are trained to round down to the nearest whole millimeter. Common factors that often contribute to measurement bias include inconsistent angulation, probe pressure, and measurement rounding. Overall, reliability findings from our study indicate that periodontal measurement error was more likely to occur with pocket depth measurements and less with recession (and attachment loss) measurements.

Because our oral health data collection methodology used NHANES protocols, we can compare our examiner reliability findings to those published from NHANES. For this current study, the calculated examiner agreement for tooth retention and untreated dental caries was consistent with what has been observed from NHANES [14, 16]. In NHANES 2003–04, examiner Kappa statistics ranged from 0.65 to 0.73 for untreated caries (0.57–0.81 in our study) and 0.94–1.00 for tooth retention (1.00 in our study). When evaluating continuously-scaled measures of periodontal disease, the overall ICCs for pocket depth was 0.61 and 0.86 for NHANES 2003–04 and 0.61 and 0.72 for NHANES 2009–10. In the current study the calculated ICCs for pocket depth were 0.79 and 0.81. In NHANES 2003–04 mean attachment loss ICCs ranged from 0.86 to 0.93 and 0.82 to 0.87 in NHANES 2009–10, whereas ICCs for attachment loss in our study were 0.87 and 0.89. Given the similarity in examiner reliability statistics for general dental caries and periodontal assessments, examiner performance observed in the current study could be considered comparable to that reported on previous NHANES oral-health studies for selected oral health measures.

The oral health assessments conducted in our current study utilized standard assessments for dental caries and periodontal status that have been used on NHANES. Incorporating these assessments and employing similar quality assurance controls should facilitate comparison of our findings to that of the US national population. Although greater reliability is certainly desirable, it is worth noting that less-than-perfect reliability does not invalidate subsequent investigations; rather, one can conclude that less-than-perfect reliability has implications for the precision of corresponding evaluations, so analyses of measurements in the lower echelon of reliability categories should be interpreted with an understanding that reliability of measurement is a concern. By highlighting areas where reliability of measurement was not high, we hope to call attention to areas where additional attention to training and calibration protocols could be expected to have the greatest impact.

## Conclusion

Overall, the quality assurance program confirmed the procedural adherence of the quality of the data collected on the distribution of dental caries and periodontal disease in MA-users. Examiner concordance was higher for dental caries but lower for specific periodontal assessments.

## Abbreviations

MA: Methamphetamine abuse
QA: Quality assurance
NHANES: National health and nutrition examination survey
CDC/AAP: Centers for disease control and prevention / American academy of periodontology
DMFT: Number of decayed, missing, and filled teeth
DS: Number of decayed coronal surfaces
ICC: Intra-class correlation coefficient
